# Treatment with oestrogen or manual separation for labial adhesions – initial outcome and long-term follow-up

**DOI:** 10.1186/s12887-018-1018-x

**Published:** 2018-03-08

**Authors:** Ellen Wejde, Ann Nozohoor Ekmark, Pernilla Stenström

**Affiliations:** 1 Faculty of Medicine, Lund University, Lund, Sweden; 2 Department of Paediatric Surgery, Skåne University Hospital, Institution of Clinical Sciences, Lund University, S-221 85 Lund, Sweden

**Keywords:** Labial adhesions, Oestrogen, Manual separation, Treatment, Long-term outcome

## Abstract

**Background:**

Topical oestrogen and manual separation are the main treatments for labial adhesions. The aim was to evaluate treatment of labial adhesions and compare the outcome of topical oestrogen treatment with that of manual separation.

**Method:**

All girls aged 0–12 years admitted to a tertiary centre for paediatric surgery for labial adhesions were included. The study design was dual: The first part was a retrospective chart review of the treatment success according to the medical charts. The second part was a cross-sectional parent-reported long-term outcome study (> 6 months after last treatment finished).

**Results:**

In total 71 patients were included and the median follow-up time for the chart study was 84 (6–162) months after treatment with oestrogen or manual separation. Oestrogen was the first treatment for 66 patients who had an initial successful rate of 62% but this was followed by recurrences in 44%. Five patients had manual treatment as their first treatment and they had a 100% initial success rate followed by recurrences in 20%. Therefore, for the first treatment course there was a final success rate of 35% for oestrogen and 80% for manual separation (*p* = 0.006). Corresponding final success rates including all consecutive treatments over the study period were 46/130 (35%) for oestrogen and 21/30 (70%) for manual separation (*p* = 0.001). The success rate for oestrogen did not differ if treatment was given in a course length of 0–4 weeks (39% success) or > 4 weeks (32% success) (*p* = 0.369).

In the parent-reported long-term outcome study the response rate was 51% (36/71).

Parents reported that recurrences of adhesions after last prescribed/performed treatment were frequent: in total 25% of patients still had adhesions corresponding to 8/29 (29%) of those whose last treatment was oestrogen and 1/9 (11%) of those whose last treatment was manual separation.

**Conclusion:**

Due to the results recurrences are common after both oestrogen and manual separations. However, the overall final outcome after manual separation seems to be more successful when compared to that of topical oestrogen treatment.

## Background

Labial adhesions are defined as when the labia minora are partly or completely agglutinated. The incidence is reported to be around 1.8% and the diagnosis occurs most frequently between 13 and 23 months of age [1]. The symptoms are related to urinary outlet obstruction [2–5] but more than 35% of labial adhesions are reported to be asymptomatic [4, 5]. If no symptoms are present, some authors recommend reassurance and a watchful wait [2] and other authors recommend treatment in order to avoid symptoms [5, 6]. Most authors recommend treatment as a result of symptoms [2, 5–7].

Topical oestrogen ointment applied to the adhesion area is often used as a first-line treatment option [4, 5, 7, 8]. Manual separation of the labia is often reported as a second-line treatment option when topical treatment fails [2–5, 7, 8] or when topical oestrogen therapy is refused by the child or parent [3]. In previous studies, the initial success rates after topical oestrogen treatment are reported to be 15–100% [2–5, 7] and recurrence rates differ between 11 and 41% [4, 5, 8]. For manual separation a recurrence rate of 15% is reported [5]. The question that still remains to be answered is which of the treatments is the best to be recommended to girls with symptoms because of labial adhesions, in terms of aspects of long-term outcome, side-effects and parental concerns with the two treatments.

The main aim of this study was to compare the outcome of topical oestrogen treatment with that of manual separation in both short- and long-term follow-up and to evaluate the families’ experiences with each treatment.

## Methods

### Patients

All girls aged 0–12 years referred to the Department of Paediatric Surgery at Skåne University Hospital from November 1999 until January 2014 because of labial adhesions were included. The Department is a tertiary centre that serves an area with 360,000 local residents with primary surgical care for children under 15 years of age. It is the sole centre for tertiary specialised paediatric surgery for all children in an area of 1.8 million residents. The health care is free for all children.

Referrals for labial adhesions came from paediatricians or the primary health care team. The indication for treatment was symptoms of obstruction, itching and redness in the local area. Exclusion criteria were absence of symptoms, treatments other than oestrogen or manual separation, or congenital malformations in the anorectal- and genitourinary tract. In total, three doctors were responsible for the treatments.

### Retrospective chart review

Patient charts with a minimum of 6 months having elapsed since the commencement of treatment were reviewed. Patients receiving treatments other than topical oestrogen and manual separation, including hydrocortisone, other creams, only petroleum ointment, and no treatment, were excluded from the study. Information about which type of treatment (oestrogen or manual separation), age at the time of first treatment, initial success and recurrences was recorded. Patients with no adhesions or recurrences reported in charts 6 months after the initiation of treatment, were registered as having had a ‘final success’. Information about treatment length with oestrogen, petroleum ointment prescribed post-treatment and documented side-effects of oestrogen and manual separation was also collected.

### Outcome and treatment definitions

Outcomes were defined as ‘initial successful treatment’ (totally resolved adhesions) and recurrence after an initially successful treatment. Only complete resolutions of adhesion were registered as successful treatments while partly separated adhesions and absence of any separated adhesion were classified as unsuccessful treatments. Recurrences were defined as complete or partly recurrent adhesions.

The endpoint was ‘final successful outcome’ which was a successful outcome after a minimum of 6 months after each treatment without any recurrences noted in the medical charts.

Treatment periods with oestrogen were therefore grouped into 1–4 and > 4 weeks. The length of oestrogen treatment was 4 weeks according to the local care programme. The cut-off at 4 weeks in the clinic and study was based on the recommended treatment length of 2–6 weeks [3, 5, 7]. Continued treatments after a pause of > 2 weeks were recorded as new treatments. The parents were shown how to apply the oestrogen cream 0.3–0.6 ml twice a day by the physician.

Treatment with manual separation was performed under general anaesthesia during 1999–2006, then using sedation and local anaesthesia in the outpatient clinic during 2007–2014. For general anaesthesia, Propofol®, Sevoran gas® and Ultiva® were used. In the procedure with local anaesthetics, Xylocain® ointment was applied on the adhesions 60 min before the procedure. Then, 15–30 min before the planned separation, midazolam (Dormicum®) 0.1 mg/ml was administered orally or rectally. Independent of the type of anaesthesia, all patients with manual separation treatment were grouped together in the analyses.

Post-treatment with petroleum ointment (Vaseline®) was defined as a documented recommendation to parents to start using petroleum ointment as soon as the adhesions were resolved, either after oestrogen treatment or after successful manual separation. The parents were shown how to apply a minimum dose of 2 ml twice a day of the petroleum ointment, for at least 1 month.

The selection of all treatments including initiation of post-treatment with petroleum ointment was at the discretion of the treating doctor. Outcome was analysed after each single treatment *and* after the first two treatments, thus taking into account the possible additional influence by the first treatment on the outcome of the second treatment.

### Parent-reported long-term outcome study

Patients whose last patient chart entry was at least 6 months prior were selected. The parents were asked through a letter to participate in the study. Those who choose to participate were given a time for a questionnaire-based telephone interview. The questionnaire focused on the parents’ answers regarding:

1. Recurrent or persisting problems with labial adhesions after the last visit to the department. 2. Their subjectively experienced convenience with the treatment or treatments graded according to a scale 1–5 (1 = most complaints, Table 1). The type of problems they had experienced were also collected. 3. They were also asked if they would recommend the same treatment to other families with the same problem or not (Table 1). The symptoms and recurrent adhesions in the parent-reported study were subjectively reported by parents and not ascertained by a doctor. Therefore they were separately reported from the retrospective chart study.

**Table 1:** Questions asked during telephone interviews with parents of patients treated because of labial adhesions at the last consultation at the Department of Paediatric Surgery, at least 6 months ago

1. Did your daughter have any persisting or recurrent problems (symptoms) with adhesions since the last visit at the department?
2. Does your daughter have adhesions at present?
3. What was the last treatment given for labial adhesions?
4a. Did you notice any side-effects from the treatments given?
4b. If so, what kind of side-effects did you experience? Breast development/rash/pigmentation/skin irritation/scarring/bleeding/pain/discomfort during separation/other (please specify)
5. How do you experience the treatments (specify oestrogen and/or manual separation) on the following score from 1 to 5?
1: The treatment was extremely complicated and inconvenient
2: The treatment was complicated and inconvenient to a fairly large extent
3: The treatment was a bit complicated and/or inconvenient
4: The treatment was not very complicated or inconvenient
5 The treatment was neither complicated nor inconvenient
6. What problems with the treatment did you experience (please specify for each treatment): Time-consuming/unclear treatment instructions/ anxiety/pain/side-effects/ discomfort touching the area/difficulties with applying the cream/other
7. Would you recommend other parents to use the treatment on their children? (please specify treatment): Yes/no/do not know

### Statistical methods

Fisher’s two-tailed exact test was used for dichotomous variables and the Mann-Whitney U-test for ranked results. A *p*-value < 0.05 was considered statistically significant. A Bonferroni-correction was done for relevant results to counteract the increased risk of type-I error. SPSS Statistics 20.0 was used for statistical calculations. A statistician designed the statistical analyses.

### Ethical consideration

The study was performed according to the Helsinki Declaration and approved by the Regional Ethical Review Board (registration number 2010/49). The data were made anonymous prior to calculations, and are presented in such a way that it is impossible to identify any single patient.

## Results

### Retrospective chart study

#### Patients and number of treatments

During the study period, a total of 80 patients were referred to the department because of labial adhesions. Nine patients were excluded because they presented without symptoms and received no treatments (five patients) or received treatments other than oestrogen and manual separation (four patients had steroids, zinc cream, or only petroleum ointment). There were no girls with anorectal or urinary-tract malformations. Thus the 71 patients who received topical oestrogen (*n* = 66) or manual separation (*n* = 5) at the first consultation were included in the study. The median age for the first treatment with oestrogen was 19 (2–86) months and for manual separation 27 (7–54) months (*p* = 0.122). The median duration of follow-up as recorded in the patient charts was 84 (6–162) months.

In summary, the patient group (*n* = 71) had a total of 130 treatments with oestrogen and 30 manual separations. Sixteen manual separation treatments (53%) were performed at the outpatient clinic, and 14 in the surgical ward under general anaesthetic.

#### Outcomes after the first and second treatments

**Fig. 1:**
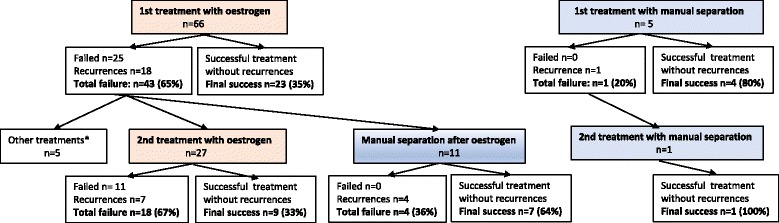
Outcome after topical oestrogen in treatment for labial adhesions in courses 1 and 2. Successful treatment was defined as initial success without recurrences at the time of at least 6 months after the last treatment prescribed. *n* = number (%)

A flowchart of outcome after both types of treatment in each patient is presented in Fig. 1. Table 2 displays recurrences and success rates after each treatment course and shows that the first treatment resulted in a final success rate of 23/66 (35%) for oestrogen and 4/5 (80%) for manual separation (*p* = 0.006). After the first course of oestrogen treatment the final success rate (35%) did not differ significantly from the final success rate after an additional second course with oestrogen after a failed first course: 9/27 (33%) (*p* = 1.000). The final success rate was 32/66 (48%) after two subsequent courses with oestrogen.

The final success rate after the first treatment using manual separation (80%) did not differ significantly from the final success rate 7/11 (64%) when manual separation was used as a second treatment after one failed oestrogen course (*p* = 0.987). Neither did the final success rate after the second treatment differ significantly whether it was two consecutive treatments using oestrogen (final success rate was 33%) or oestrogen treatment followed by manual separation (64%) (*p* = 0.147). One patient had consecutive treatment with manual separation resulting in a final successful outcome (100%) (Table 2 and Fig. 1).

**Table 2:** Initial success rate, recurrence rate and comparison of final success rate after first and second line treatments with oestrogen and manual separation

	Patients	Initial success rate	Recurrences	Final success rate	Final success rate *p*-value^a^
First treatment: oestrogen	66	41 (62)	18 (44)	23 (35)	*0.006*
First treatment: manual separation	5	5 (100)	1 (20)	4 (80)	
Second treatment: oestrogen after oestrogen	27	16 (60)	7 (44)	9 (33)	0.147
Second treatment: manual separation after oestrogen	11	11 (100)	4 (36)	7 (64)	
Second treatment: manual separation after manual separation	1	1 (100)	0	1 (100)	

^a^Fisher’s exact test, two tailed , n (%)

#### Outcomes after all treatments

When comparing outcomes after all treatments over the study period including 130 oestrogen treatments and 30 manual separations, the initial success rate was higher for manual separation than for oestrogen (*p* <  0.001) but the recurrence rate did not differ significantly (*p* = 0.263). In the end, the final success rate was significantly higher for manual separation (70%) than for oestrogen (35%) (Table 3).

**Table 3:** The total numbers and results of treatments with oestrogen and manual separation respectively. n(%)

Treatment	Total number of treatments	Initial successful treatments	Recurrences	Final success rate	Final success rate *p*-value^a^
Oestrogen	130	78 (60)	32 (41)	46 (35)	< 0.001
Manual separation	30	29 (97)	8 (28)	21 (70)	
Total	160	107 (67)	40 (37)	67 (42)	

^a^Fisher’s exact test, two tailed

#### Outcomes after different treatment lengths of oestrogen

The lengths of oestrogen treatments were documented in the charts with 85 (65%) of the 130 treatment courses with oestrogen. The median length of treatment was 4(1–12) weeks. There was no difference in outcome success when comparing treatments of 0–4 weeks with > 4 weeks (Table 4).

**Table 4:** Comparison of outcome after different treatment lengths with topical oestrogen: 1–4 weeks compared with > 4 weeks.

Treatment length	Total number of treatments	Number of initial successful treatments	Number of recurrences	Final success rate	Final success rate *p*-value^a^
Oestrogen 1–4 weeks	48	24 (50)	5 (21)	19 (39)	0.369
Oestrogen > 4 weeks	37	19 (51)	7 (37)	12 (32)	
No information	45	35 (78)	20 (57)	15 (33)	
All	130	78	32	46 (35)	

^a^Fisher’s exact test, two tailed The final successful outcome is compared statistically *n* = number (%)

#### Post-treatment with petroleum ointment

Treatment with petroleum ointment after treatment success was prescribed in 14/130 (11%) of oestrogen treatments and in 18/30 (60%) of manual separations (*p* <  0.001). The recurrence rate after an initial successful treatment with oestrogen with additional petroleum ointment treatment was 4/11 (36%), which did not differ from recurrences 28/67 (42%) without additional petroleum ointment treatment (*p* = 0.967). Neither was there any statistical significant difference in recurrences in 4/18 (22%) versus 4/11 (36%) after manual separation with or without additional treatment with petroleum ointment, respectively (*p* = 0.433).

#### Side-effects

Four patients (6%) had experienced side-effects of oestrogen treatment: two cases of breast gland hypertrophy, two cases of local redness and irritation and one experienced both symptoms. All side-effects occurred within the first 4 weeks of oestrogen treatment. No side-effects or complications from manual separation were reported in charts.

### Parent-reported long-term follow-up

#### Response rate, treatments and symptoms after last consultation

The response rate in the parent-reported long-term parental report follow-up study using telephone interviews was 36/71 (51%). At the time of the interview, the median follow-up time after the last consultation was 53 (6–144) months. The parents interviewed had experiences from a total of 48 treatments with oestrogen and/or manual separation. The last treatment given was oestrogen in 27 (75%) and manual separation in nine (25%) patients. Among those who did not participate in the interview 23 (66%) cited oestrogen and 12 (34%) cited manual treatment as their child’s last recorded treatment. Overall, the median number of all who received treatments did not differ between patients of participating parents (median 2, range 1–10) and non-participating parents (median 2, range 1–6) (*p* = 1.000).

Symptoms of labial adhesions at any time after the last consultation at the department were reported by in total 13/36 (36%) and symptoms reported were rash, pain, obstruction and infection. Any symptoms after the last treatment with oestrogen were reported by 11/27 (41%) and after manual separation by 2/9 (22%) (*p* = 0.438). At the time of the interview, parents of nine (25%) children reported that their child still had adhesions of whom 8/27 (29%) had oestrogen and 1/9 (11%) had manual separation as the last treatment (*p* = 0.393).

#### Scorings and experiences

**Fig. 2:**
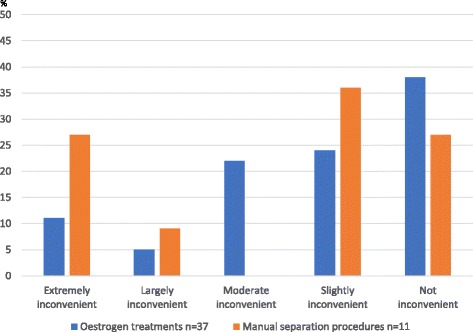
The distribution of the subjective experience of treatment scored 1–5 by parents (see Table 1 for definition of the score)

The 36 parents scored their experiences from the 48 treatments with oestrogen (*n* = 37) and manual separation (*n* = 11), on a scale of 1–5 (1 = worst, see Table 1). The reported median score for each treatment, both oestrogen and manual separation, was 4 (1–5) without any statistical difference (*p* = 0.434) (Table 5). The distribution of scores is displayed in Fig. 2 and the distribution did not differ statistically (*p* = 0.075). Comments from the experiences with oestrogen treatment were problems with frequently recurrent adhesions, and discomfort from touching the labial area for both parents and the patient (*n* = 22). Applying the oestrogen cream was reported to be more difficult when the patient stopped using diapers and the procedure was no longer natural (*n* = 8). Parents were also worried about eventual side-effects from treating their daughters with hormones (*n* = 16). Comments on manual separation at the outpatient clinic were pain and discomfort during the procedure (*n* = 3). When performed in the surgical ward there was concern over possible risks and discomfort with general anaesthesia (n = 2).

**Table 5:** Results from telephone interviews with parents of 36 patients with experiences from 48 treatments with oestrogen and/or manual separations

Treatment (n)	Experience Score 1–5 Median (range)	*p*-value^b^	Would not recommend the treatment to others/do not know	Would recommend the treatment to others	*p*-value^c^
Oestrogen *n* = 37	4 (1–5)	0.434	5/2	30 (68)	1.000
Manual separation^a^ *n* = 11	4 (1–5)		0/3	8 (73)	

^a^ 5 were under local and 6 under general anaesthesia

^b^ Mann Whitney

^c^ Fisher’s exact test two tailed comparing would not recommend the treatment to others/do not know with would recommend the treatmentReported inconvenience with treatments was scored 1–5 (1 = very inconvenient, 5 = no problems according to Table 1). The distribution of the scored experience is visualised in Fig. 2. n (%)

Overall 69% of parents reported that they would recommend the treatment to other parents. The rate of recommendations did not differ between oestrogen and manual separations (Table 5).

## Discussion

### Summary

In the retrospective follow-up manual separation turned out to be a more successful treatment for labial adhesions compared to topical oestrogen both according to the outcome of the first treatment and in analyses of all treatments. The length of treatment with oestrogen did not change the outcome, nor did the post treatment with petroleum ointment. Also, according to parent reports in the long-term follow-up, there was a trend towards a better outcome for manual separation but recurrences were common after both treatments both in the retrospective chart review and according to the parent reports. Two-thirds of the parents would recommend either of the treatments to others.

To the authors’ knowledge, this is the first study to present long-term studies on labial adhesions with parental interviews.

### Retrospective chart study: oestrogen treatment

In the retrospective part of the study, the initial success rate after oestrogen treatment courses was 60% but a final success was only achieved in 35% of the treatments since the recurrence rate was high. In previous studies on outcome after oestrogen treatment, both lower (15%) and higher final success rates (100%) were reported [6, 8]. A retrospective chart review, similar to ours, showed a slightly higher rate of initial successful separation (71%) but a similar recurrence rate (35%) [4]. In a smaller study, the initial success rate was also slightly higher (67%) but the recurrence rate was lower (11%) [5]. Speculating, differences in successful outcome could be explained by different thickness of adhesions [7] or different compliance to treatment maybe because of cultural differences in accepting a prescribed hormonal treatment, or fear of the hormonal side-effects of oestrogen.

Differences in recurrence rates are slightly more difficult to explain but, speculating, they could depend on post-treatment regimens. However, the influence of hygienic factors on recurrences have previously been evaluated without findings of any strong predictors [7]. In our study the post treatment with petroleum ointment could not be proved to protect fully against recurrences. However, the use of petroleum ointment after manual separation was significantly higher than after oestrogen treatment, which could be considered to be one of many possible reasons for the lower recurrence rates after manual separation.

### Retrospective chart study: length of oestrogen treatment

When evaluating the duration of oestrogen treatments, the results in our study support that most adhesions that will ever respond to oestrogen treatment do so within 4 weeks. Comparing our results to one previous retrospective study that included oestrogen treatment for only 2 weeks, the referred study reported an initial success rate of 67% [5] which is slightly higher than the result (50%) in our study of 1–4 weeks’ treatment. Another study, with an average treatment length of oestrogen of 2.2 months, reported both a similar initial success rate and recurrence rate as ours [4]. However, there is also one report which, after 4 weeks of treatment with topical oestrogen, only had a final success rate of 15% [8]. On the other hand, one prospective study with closely monitored treatments reported full success (100%) with a treatment length of 2.4 months which included a careful follow-up, prophylactic treatment and meticulous hygiene [6]. The diverse results on different treatment lengths indicate that the duration of the treatment may not be the single factor to influence the outcome.

### Retrospective chart study: manual separation

Regarding manual separation, our study revealed a high initial success rate, a lower recurrence rate and also a higher final success rate (70%) compared to oestrogen treatment. Previous studies on manual treatments report the same high initial success rates as ours [5, 8, 9]. Also, in line with our study, a recurrence rate of 26% and accordingly a final success rate of 74% has been reported for patients having manual separation after failed medical treatment with oestrogen [4]. In one study, the final success rate of manual separation used after one failed oestrogen treatment course was 86% but if used as first-line treatment with 5 days of post-treatment with topical oestrogen, the final success rate reported was 100% after 3 months [5]. A similar high final success rate (100%) after manual separation as first-line treatment was reported in a prospective smaller study with only eight patients. Those patients received post-treatment with gentamicin ointment and careful washing during the 6 months of follow-up [10]. Once again, there seem to be important additional factors, other than which treatment is used, that influence the success rates.

### Parent reports in telephone interviews

In the parent interviews, as many as one-third of the patients reported having had continuous problems with recurrences of the adhesions after the last consultation. To the author’s best knowledge there is no similar study to compare this outcome with. When grading the parental experiences, the parents on the whole seemed quite pleased with the treatments grading the treatments with oestrogen and manual separation as both 4 (1–5) but as viewed in Fig. 2 more parent reports on oestrogen scored in the middle of range while experiences from manual separation tended to be more diverse. Concerning discomfort another study reported, in line with our results, mainly mild to moderate discomfort during manual separation under local anaesthetic [11]. However, contrary to this, only 70% of the parents in our study answered that they would recommend the treatment to others. Possible reasons for hesitation in recommending treatment might be the reported concerns about the pain and discomfort, and also the risk of recurrences and maybe side-effects.

### Side-effects and indication for treatment

In total, 6% of those treated with oestrogen reported or had documented side-effects while none were reported/documented by those who underwent manual separation treatment. In the previous literature, short-term side-effects from topical oestrogen, such as breast development, vaginal bleeding, local skin irritation, rash and vulvar pigmentation were reported to be higher than our result, and present in 6–22% [2, 4–6, 8]**.** Long-term side-effects after childhood are unknown, and there are no reports on side-effects after manual separation. The unknown long-term side effects of oestrogen must be considered when taking the decision to treat or not, and what treatment to use. Risks of topical oestrogen in adult women, e.g. with breast malignancies, are currently being discussed [12–14] but there is no longitudinal study on children receiving topical oestrogen. Neither is there any longitudinal study on long-term side effects of treatment with manual separation.

Furthermore, there are no studies reporting on spontaneous resolution of adhesions and what happens in the long term if no treatment is prescribed. But in the light of that the final success rates after recurrences turn out to be quite low, and because of the parental hesitation to recommend the treatment, treatment should only be advocated if a strong indication is present, such as obstruction leading to urinary infections, or intense rash.

Overall, the chances of reaching a complete and permanent resolution of adhesions seem to increase if adhesions are thin and the treatment is combined with post-treatment prophylaxis and compliance is assured by close follow-up for several months [2, 3, 5–7, 10].

### Limitations and strengths

One strength of the study was the number of oestrogen treatments which was larger than in previous studies [4–6, 8] and the total number of manual separations is similar to previous studies [4, 5]. Criticism could be raised that each treatment was also analysed separately because there could be additional effects from previous treatments. However, since the overall outcome did not differ from the outcomes after subsequent treatments, we considered the results of the total number of treatment as reliable.

There might be a selection bias because all patients were referred from general practitioners or paediatricians, and the adhesions might have been more severe/thick than in those patients not needing a referral. Since possible treatments before referrals were not always documented in admission notes or in charts and the chart study was retrospective, this important knowledge is lacking in the study. Another aspect that might lead to a better outcome for manual separation is that the initial successful outcome after manual separation could be because the personnel at the surgical ward were accustomed to similar routines. To perform manual separations well and to make the child and its parents confident with the treatment, the technique demands personnel that are familiar with the procedure. When comparing initial success rates after manual separation between different departments one needs to take into account that routines and experiences among the personnel could differ.

The main weakness with the study was the limited number of patients who had treatments with manual separation. Another weakness with the retrospective chart study was the inclusion of some inaccurate and incomplete information because of the retrospective design. In a future prospective study definitions stated in advance, regarding thickness of adhesions, various grades of successful treatment and compliance would increase the reliability considerably. The main weakness with the parent-reported study was the limited response rate (51%). It is debatable whether the answering parents were representative of all, or if only those with problems chose to get in contact as a result of the study. Then, to really understand long-term outcomes in the treatment of labial adhesions, longitudinal studies with a regular follow-up after 1 or 2 years would be valuable.

In the end, clear guidelines based on randomised studies would be very helpful in the choice of treatment of labial adhesions. A prospective randomised trial with accurate measures, both of different treatments and without any treatment for labial adhesions, would give more exact and reliable results. There is an urgent need for such a study.

## Conclusion

Labial adhesions in young girls seem to be more effectively treated with manual separation than topical oestrogen, but the long-term recurrence rates after both treatments are high. Randomised studies with longitudinal long-term follow-ups over childhood are needed to create strong evidence for future treatment guidelines for labial adhesions.
